# Readiness for the Realities of Internship: An Ethnographic Study Using Legitimation Code Theory

**DOI:** 10.5334/pme.1802

**Published:** 2025-09-26

**Authors:** Stuart Redvers Pattinson, Hans Savelberg, Anique Atherley

**Affiliations:** 1 Unit for Undergraduate Medical Education (UUME), School of Clinical Medicine, Faculty of Health Sciences, University of the Witwatersrand, Johannesburg, Gauteng, South Africa; 2 School of Health Professions Education (SHE), Maastricht University, Maastricht, Netherlands; 3 Department of Nutrition and Movement Science, School of Health Professions Education (SHE), Faculty of Health, Medicine and Life Sciences, Maastricht University, Maastricht, Netherlands; 4 Office of External Affairs, Ross University School of Medicine, Barbados

## Abstract

**Introduction:**

Many newly qualified doctors struggle to cope with the challenging transition to internship, raising a concern over whether they are adequately prepared with the competencies they need for legitimate practice. This study aimed to explore the reality of the internship role, the types of competencies that form the basis of achievement in this context, and the perceived readiness of newly qualified doctors to demonstrate these competencies.

**Methods:**

A qualitative study, using focused ethnography, was undertaken in a regional public hospital in Johannesburg. 15 first year internship doctors volunteered to take part. Data was gathered over a seven-week period through participant observation and shadowing and informal conversations. Semi-structured interviews were recorded with 13 of the participants to further explore emerging themes. The data were analysed using a reflective thematic analysis with additional analysis using the Legitimation Code Theory (LCT) specialisation dimension coding framework.

**Results:**

Interns are the ‘engine room’ of the hospital, facing long hours and the responsibility for a high workload. Some interns cope well, while others become overwhelmed, negatively impacting their wellbeing. Competency in teamwork, organization, efficiency, and communication provides the basis for legitimate practice, all attributes not highly valued by the undergraduate curriculum.

**Discussion:**

To thrive, newly qualified doctors need to demonstrate both confidence in their clinical knowledge and skills as well as the attributes that form the basis of achievement in internship. To achieve this we need holistic competency based medical education that meaningfully values the personal and social competencies that are critical for legitimate practice.

## Introduction

The transition from student to doctor can be a challenging one [1]. Some graduates adapt positively in the face of this challenge, applying what they have learnt and growing in competence and confidence [2]. Others experience this transition as abrupt and overwhelming, finding themselves unprepared for the hurdles they are expected to overcome. This can lead to anxiety, stress, loss of confidence, decreased wellbeing and burnout [3,4]. For a newly graduated internship doctor (intern) to thrive in their new context they need to develop legitimacy, which involves displaying the competencies – knowledge, skills and attitudes – which are considered the basis of achievement in that setting [5]. In order to adequately prepare graduates for practice, we need to ensure that these competencies needed for legitimate practice in internship are valued by the undergraduate curriculum. This can potentially be achieved through well aligned Competency Based Medical Education (CBME) [6], yet what these competencies should actually look like in the South African setting remains underexplored [7,8]. A scoping review on CBME literary conversations found that only 0.5% of first authors were from Africa [8] and research into post-graduate CBME in South Africa has highlighted both the lack of local research and the importance of curriculum reform that is contextually relevant [9]. In 2014, the Health Professions Counsel of South Africa (HPCSA) adopted the AfriMeds framework in order to provide core competencies for undergraduate students as a framework for curriculum development [10]. While the AfriMEDS curriculum framework could produce technically skilled practitioners, its effectiveness in nurturing well-rounded medical professionals that can adapt to new challenges has been questioned [10]. Our own previous research, exploring the perceptions of first year interns, revealed a clash between what graduates felt well prepared for and the expectations of the internship role [11]. There is a need for CBME curriculum reform incorporating more holistic educational strategies that considers the legitimate knowledge, skills and attitudes needed for a new junior doctor to thrive in the workplace [10].

To understand how legitimacy is gained, the ‘rules of the game’ that facilitate an individual’s integration into the medical environment in a manner that is valued and rewarded by the culture must be uncovered [5]. We need a nuanced understanding of the challenges interns face and the types of competencies that are needed to overcome those challenges. We should also know whether graduates feel they are equipped with these types of competencies when starting medical practice. In their narrative review on the preparedness of medical graduates, Padley et al. [12] found that most studies were retrospective, asking graduates to reflect on their experience of the transition to work. While this is a valuable source of intern’s perspectives, relying on recall can be unreliable and correlation with actual performance is uncertain [13]. There is a need for observational research designs, of which there are few [14], to provide a deeper understanding of the contemporary intern experience. Observational studies provide a rich understanding of the reality of practice and routines of everyday activities [15] and immersion in the complex clinical setting can reveal the hidden aspects of the culture of the internship experience. Atkinson et al. [15] suggest that “there is a need for close and prolonged ethnographic engagement with medical students, junior doctors and other health care professionals to explore and change the many assumptions that exist in these settings and which are taken for granted.” Observational methods can help ascertain the factors impacting students’ ability to successfully transition to internship, allowing them to perform the job competently as a valued part of the team while maintaining their well-being [16].

In order to answer both the calls for a more nuanced understanding of what competencies provide legitimacy in the South African context and more observational research exploring the transition to internship, this study aimed to use focused ethnography to explore the reality of the internship role, the types of competencies that are valued by the community, and the perceived readiness of newly qualified internship doctors to demonstrate these competencies. In doing so, we hope to make recommendations for practice and future research, including informing more holistic undergraduate medical CBME curricula development going forward.

## Study Aim

This study aims to answer the following research questions:

1. What is the role of an internship doctor and what challenges do they face?
2. What competencies are legitimised by the community in which interns practice?
3. To what extent do interns feel they are equipped with these competencies going into internship?

## Methods

### Study Design

This is a qualitative, focused ethnography [17]. Ethnography allows for the study of social interactions, culture, behaviours and perceptions within a community, providing data on ‘what actually happens’ in a particular setting, rather than what people ‘say’ happens. The latter may be affected by social desirability bias, selective perception, and poor recall among participants [18]. Focused ethnography differs from traditional ethnography with more intense, targeted data collection via intermittent visits over a shorter time frame, with narrower aims involving a specific issue and context [17]. This made focused ethnography a suitable and more feasible alternative to prolonged ethnography for our research aims which involved interns in a clinical setting where they complete relatively short rotations and regularly move between departments and facilities. Focused ethnography is “a legitimate and well-suited way to explore specific fields with specific characteristics, such as medical education” [17]. Immersion in the workplace setting allows for rich and holistic insights into social practices, including people’s views and actions, and the nature and culture of the setting [18], uncovering what is often unseen or taken for granted [19]. Observation of the actual work of an intern can show what types of competencies are used to do the job, and informal discussion can ascertain interns’ perceived work readiness to meet these competencies.

### Theoretical Framework

In this study we used Legitimation Code Theory (LCT) as a grounding framework in a fully theory-informed inductive approach [20] to understanding the basis of legitimacy in the internship context [5]. The need to establish legitimacy before learning within a community of practice has been established in medical education through the work of Lave and Wenger [21]. Building on this understanding, the Specialisation dimension of LCT facilitates the specific exploration of the basis of achievement underlying practices, making explicit what knowledge, skills and attitudes are valued within the medical internship context. This is achieved through analysing the epistemic (ER) and social relations (SR) within qualitative data. Epistemic relations (ER) denote the value being placed by the community of practice on the possession of specialised knowledge and social relations (SR) indicate the value being placed on ‘ways of being’ – personal and social attributes such as resilience and teamwork. This approach allows for value placed on professional attributes orientated towards ‘what you know’ (specialised knowledge) to be compared to those orientated towards ‘who you are’ (personal attributes) in establishing legitimacy. To date, the specialisation dimension of LCT has been used in higher education research to show that if students’ dispositions clash with the basis of achievement of their context, they will struggle to learn and succeed [5]. Other dimensions of LCT are Semantics and Autonomy, which focus on the context-dependence and complexity of practices and the relations among different sets of practices. We will focus on the specialisation dimension of LCT to answer our second and third research questions and reveal the types of competencies that form the basis of achievement in internship and need to be demonstrated by interns to be legitimate participants [22].

### Setting and Study Population

Newly qualified junior doctors in South Africa must complete a mandatory two-year internship at an accredited training facility. In the South African clinical context, interns face high workloads, low doctor-to-patient ratios, long work hours and limited resources [23]. High levels of burnout among South African interns [24,25] suggest that many graduates in this context find the transition to internship a threat to well-being and professional progression.

The setting for this study was a regional mother and child public hospital in Johannesburg, South Africa. This hospital was selected as it provided accessibility to participants and purposeful sampling [17]. First-year internship doctors were purposively sampled from the Obstetrics and Gynaecology (O&G) and Paediatrics departments. A previous study with 32 interns working in a wide range of South African hospitals [11] suggested that the rotations and level of this facility would provide a fair representation of the intern experience. This hospital has 15–20 first-year internship doctors from South African universities rotating through both the O&G and Paediatrics departments. The principal researcher (SP) was an outsider, not having ever worked or taught at this facility. He introduced himself to the whole clinical department and explained the study during the departmental morning meetings. In these meetings all first-year interns were invited to take part in the study. Willing participants were invited to approach the researcher in person or via email if they were interested in signing up. A total of 15 interns (8 female and 7 male) volunteered to participate in the research and provided written informed consent. Data collection occurred during the participants’ second internship rotation, meaning all participants had experience in both Paediatrics and O&G at the time of data collection.

### Data Collection

Data were gathered by the principal researcher (SP) through participant observation, shadowing and informal conversations. SP visited the hospital 28 times over seven weeks, including two night shifts and two weekend shifts, spending approximately 180 hours at the research site. Observations continued until sufficient information power was established to answer the research questions [26,27]. The researcher took on the role of complete observer [18], but demonstrated reflexivity by describing his ideas and experiences and the possible impact those might have on the findings in the field notes.

Keeping the research questions in mind, SP made detailed field notes while observing daily routines and interactions in the wards, outpatient clinics, operating theatre, the emergency department, and when consulting other departments. In each of these settings, SP would introduce himself, explain the research and obtain verbal consent to continue the observations while focusing on the research participant. Informal conversations occurred between the researcher and the participants during suitable moments, such as walking between wards and in the tearoom. The tearoom proved to be a valuable place to spend time and talk with interns as they stopped for a bite to eat and to debrief about their day. Semi-structured interviews were recorded with 13 participants (7 female, 6 male) near the end of the data collection to further explore the emergent issues from the observations toward answering our research questions.

### Data Analysis

Field notes and audio recordings of the semi-structured interviews were transcribed verbatim and pseudonymised by the principal researcher. These were uploaded onto *Atlas.ti* and shared with the research team, who then undertook a reflexive thematic analysis based on Braun and Clarke’s suggested criteria [28,29]. SP and AA familiarised themselves with the data and then generated initial codes. As these codes were generated, they were assigned as having strong (+) or weak (–) epistemic and social relations in an iterative process alongside the data analysis using a translation device (Table 1) devised by the authors. The translation device links the strengths and weaknesses of epistemic and social relations with indicators of their manifestation in the dataset [5].

**Table 1:** Translation Device.


EPISTEMIC AND SOCIAL RELATIONS	INDICATOR	EXAMPLE

ER +	Possession of detailed clinical knowledge is valued as the basis of achievement	“we get rewarded in med school for doing well academically and then we think we’re great, we’re going to be great doctors.” P5

ER –	Content knowledge is not valued or rewarded	“the knowledge, like, the raw, like book learning, I’ve probably gotten more of that then it seems, like, I need for the job.” P7

SR +	Personal and social skills form the basis of legitimate practice	“So, number one, without a doubt… is teamwork. You have to be a team player.” P12

SR –	Personal and social attributes are downplayed	“Soft skills are so important but not really taught or valued as undergraduates.” P5


Based on the overall strength (+) and weakness (–) of the epistemic and social relations, codes could then be labelled as one of the following:

- Knowledge codes (ER+, SR–): emphasis on specialised knowledge over personal attributes (what you know) as the basis of legitimacy
- Knower codes (ER–, SR+): emphasis on personal attributes over specialised knowledge (who you are) as the basis of legitimacy
- Elite codes (ER+, SR+): possessing both specialised knowledge and being the right kind of knower is the basis of legitimacy.
- Relativist codes: (ER–, SR–) where neither is emphasised and anything goes.

This was a qualitative process based on discussions within the research team. Initial themes were then generated, which were then reviewed and refined by the whole research team, before being defined and named. The overall findings are illustrated by plotting themes on a Specialisation Cartesian plane created by two perpendicular axes that represent the continua of strong to weak epistemic and social relations of their constituent codes. This process provides an overall visual representation of the underlying ‘rules of the game’ within the internship context, illustrating what is legitimised and forms the basis of achievement. This analysis uncovers code clashes and matches (conflicts or alignment between different codes in a single context) and code shifts (where the basis of achievement changes between different contexts, such as medical school and internship). Identifying code clashes and code shifts can be valuable in understanding why some interns thrive while others simply survive.

### Ethical Considerations

This study was approved by the Maastricht University Faculty of Health, Medicine and Life Sciences Research Ethics Committee (Approval Number: FHML-REC/2024/019). Permission to conduct the research was granted by the hospital where the research took place (NHRD REF NO: GP_202311_077). Written informed consent was obtained from all participants before participating in the study. It was stressed that participation was voluntary and that the participants could, at any time, withdraw their participation. No participants withdrew their participation.

### Reflexivity

The research team consisted of the principal researcher (SP) who is pursuing a PhD in Health Professions Education and two experienced educationalists and researchers (AA and HS). The principal researcher (SP) is a lecturer involved in undergraduate medical training and became interested in this topic out of a concern for how many graduates seemed to be struggling with the transition to internship. He brought his own ideas, experiences and biases to the study, and this, along with reflections on the interactions and relationships between the observer and the participants and the impact this may have had on the results, was discussed extensively within the team. He is not involved in the internship programme in any way and does not work or teach at the hospital where the research took place. AA is a medical educator with research interest in transitions during medical education. She has conducted transition-focused research and acknowledges preconceived perspectives which were shared appropriately during analysis discussions with the team. As a teacher and curriculum developer, HS noticed the importance of students’ intrinsic motivation to study. As an educational scientist, he wants to understand design components that facilitate intrinsic motivation. In this context, he is aware that being qualified to perform the medical treatments is not sufficient and that competencies in the social and personal domain are required.

The study took place after the interns had completed three months of internship, allowing time for them to have settled in but still have recent recollection of the transition. Interns are already frequently observed, being monitored regularly by peers, superiors and other health care professionals, so the experience of being observed should not have been novel [30]; however, participants could withdraw at any time.

## Results

We identified three key findings from the data. First, internship is a very challenging time that some individuals cope with better than others. Second, knower code attributes with stronger social relations (SR+) such as teamwork, organisation and efficiency are valued in successfully executing the intern role. Finally, in transitioning to practice, students had to demonstrate elite code competencies (strong epistemic and social relations; ER+, SR+) in order to successfully navigate the shift between the knowledge code basis of achievement in the medical school context (ER+, SR–) and the knower code basis of achievement in internship (ER–, SR+).

### Finding 1: Thriving or surviving – the reality of the intern role

#### Challenging intern role

Interns play a vitally important “engine room” (P11) role in the hospital. Their responsibilities are varied and essential to the delivery of patient care, and yet, the role was hard to define, as expressed by Participant 11:

*“The role of an intern is… you’re a doctor, you’re a counsellor, you’re an admin person, you’re a phlebotomist, you are a receptionist, you are a porter very often, you are the note taker, you are the organiser of the group. And… most of the time you’re*, *like, taken for granted as well.” (P11 – interview)*

The start of internship was ‘really like jumping into the fire pit’ (P7) and constantly having to adapt to new working environments was a significant obstacle. Participant 6 described this experience:

*“There’s certain things that they’re just like, ‘Oh, usually the interns do this, the interns know how to do this.’ And you’re like; ‘I don’t know how to do that, I just started!’ So, just that constant flow of work and every day is, like, 20 new things that you’ve never done before and you’re like, OK, well, just get on the bandwagon and do it, because otherwise you’re just going to drown in the work.” (P6 – interview)*

The greatest challenge that came with this role was the sheer number of patients the interns had to see and the amount of ward work and administration that needed to be done. The patient load was significant, the hospital was understaffed and resources were limited. Participants worked extended hours and had frequent, long, overnight and weekend calls that were ‘taxing emotionally and physically’ (P2). Completing the work was ultimately their responsibility, a big shift from being a student, and it was much more work than they expected:

*“I thought I knew, like, how to be an intern, but that day I was so, like, shocked at the pace that you need to work at… interns are doing, like, 50 million things behind the scenes and that you didn’t ever figure out as a student.” (P11 – interview)*

#### Thriving versus surviving

Some interns appeared to be coping better in the face of these challenges than others. For some internship was ‘exciting’ and ‘not so bad’ while others were ‘anxious’ and ‘overwhelmed,’ with constant tiredness and fatigue affecting their work and personal lives. As expressed by participant 7, they felt like they were are in a cycle of work, recovery, sleep and work with little time for anything else:

*“I found it quite difficult, like, I was quite overwhelmed a lot of the time. I felt like I didn’t have, like, mental capacity to do anything besides, like, work, come home from work, and recover.” (P7 – interview)*

Some participants seemed to generally leave work at a reasonable time, rest a bit on call and enjoy things outside of work while others really struggled to maintain a work-life balance. This was very disappointing for many interns who had an expectation that they would be able to enjoy more free time once they didn’t have to study all the time, as illustrated by participant 11:

*“It is more difficult than being a student. Ironically, you would think that being a final year student would be the worst time, but it gets harder and you didn’t think that. Like, if I think about my partner and I, it’s most probably the least we’ve ever… we barely see each other nowadays.” (P11 – interview)*

The reality is that the internship role is important and demanding, with many challenges relating to long hours and the new responsibility for a very high volume of work. Some participants appeared to struggle with this role, while others seemed to have the competencies needed to cope well with the challenge. The next section explores what those legitimised competencies were.

### Finding 2: Efficient, organised, team player – how to thrive in internship

#### Organisation and efficiency

To thrive in this challenging environment interns needed to demonstrate certain valued competencies that formed the basis of achievement in this context. The attributes needed to succeed represented stronger social relations (SR+), including efficiency, organisation, teamwork and interpersonal skills. With the demanding workload, organisation and efficiency were some of the most important competencies (stronger social relations: SR+), as highlighted by Participant 13:

*“I’d say a good intern would be someone that is hard working*, *efficient…. efficiency really plays a huge role. If you are able to finish up your work quickly, but doing it correctly, it makes a huge difference. You don’t want to be someone who’s struggling with everything… so, definitely efficiency.” (P13 – interview) (SR+)*

The interns that seemed to thrive in the clinical team were well organised, had all the paperwork and results on hand, and could multitask, documenting and effecting the management plan during ward rounds. These thriving interns knew the patients well and anticipated what needed to be done, significantly expediting the patient’s management. They planned their day effectively, knew how to prioritise tasks and were confident in their ability to get through the work. These skills allowed them to adapt well to the unpredictable nature of the work day (stronger epistemic and social relations – an elite code: ER+, SR+).

*Interns need to be able to handle a heavy workload, significant time pressure, be efficient and organised in getting the work done and ready for their responsibilities or task at hand to change at the drop of a hat. (P11 – informal conversation) (SR+)*

Inefficient interns (weaker social relations (SR–) got flustered, often left late and rarely rested on call, perpetuating the cycle of constant tiredness.

#### Collaboration

No matter how organised and efficient an intern was, however, it was not possible to manage it all on their own. Teamwork was a critical part of the job and the interns coping well were the ones who functioned best in a team environment. They needed to be able to collaborate, communicate effectively, be willing to help others and also know when to ask for help themselves (stronger social relations: SR+). Participant 12 described teamwork as the most important skill for an intern to have:

*“So, number one, without a doubt… is teamwork. You have to be a team player. If you’re not a team player you really, really will struggle. And when I say you’ll struggle it… sure, I mean, you can fly solo and think you can handle everything, but after my experience in obstetrics, when you’re being called in three different wards at the same time, then you start to realise, the main thing, I think, is that you need to be a team player.” (P12 – interview) (SR+)*

Communicating effectively was a significant part of establishing legitimacy within the team (strong social relations: SR+). The ability to communicate in a concise and persuasive manner was essential when presenting on ward rounds and consulting other disciplines. When it came to constantly having to adapt to new working environments, the interns who could quickly build good working relationships with new teams were the ones who dealt with it the best.

*Different interns have different approaches. Some walk into wards greeting the nursing staff, learning people’s names, others keep a low profile. Some units clearly have a better team rapport than others. (Field Notes)*

#### Clinical knowledge

While the interns were often the primary contact with the patient, they were not generally the ones responsible for decision-making. The supervision was very good and interns were very rarely left to create a management plan on their own. On ward rounds, the ability to demonstrate specialised medical knowledge was expected of medical students and registrars much more so than the interns, who generally got asked more practical questions relating to the progress of the management plan. The overall result was that, while the possession of clinical knowledge was important for the participant’s own confidence, it formed a surprisingly small role in establishing legitimacy as an intern within the team (weaker epistemic relations: ER–). This dynamic was summed up by Participant 5:

*“Not so much in terms of the knowledge, I mean, the funny thing for me with internships so far… it feels to me like interns, kind of from an academic point of view, being in an academic setting, we kind of fly under the radar a bit.” (P5 – interview) (ER–)*

While the interns who seemed to be succeeding generally demonstrated elite code competencies (ER+, SR+), it was specifically the knower code (ER–, SR+) attributes which were the greatest determinants of legitimacy within the internship setting. Personal attributes formed the basis of achievement and interns who didn’t demonstrate these attributes could find it difficult to cope.

### Finding 3 – Readiness through self-directed learning – balancing the demands of medical school with preparing for internship

#### Self-directed learning

Some of the participants perceived themselves to be generally quite well prepared for the role. This readiness, however, came from being self-motivated and self-directed in seeking out clinical experiences:

*There was little pressure or expectation to get involved with patient care… so clinical experience came down to individual motivation and willingness and keenness to get involved. (P4 – informal conversation) (SR+)*

Graduates who did not actively work on the skills valued in internship could find the transition to practice to be a very steep learning curve as they realised they were not actually prepared with the skills they need for legitimate practice. A conversation with participant 9 helped shed light on why a student may find themselves in this position:

*The participant also suggested that they need to spend more time shadowing interns in preparation for the role. When asked why they don’t shadow interns more, he indicated that they always have some sort of high stakes assessment coming up, often very theory heavy, that they had to pass to be able to graduate, so that would always take preference. As a student, their primary goal was to pass and they needed to do well in those assessment to get to internship in the first place, which they would have to worry about and figure out once they got there. (P9 – informal conversation)*

#### Undergraduate curriculum

Spending more time gaining the experiences needed to be ready for internship often meant resisting the pressure exerted by multiple high stakes theory assessments that rewarded individual academic knowledge and encouraged seeking out study time and tutorials over clinical experience (stronger epistemic relations: ER+). This was summed up by participant 6:

*“There was a mismatch between, almost, what the hospitals wanted and what the university wanted, because you needed to pass an exam at the end of the day and to do that you needed to study. So a lot of people would choose, either I’m going to go home early and study or I’m going to be at the hospital. And I think the idea is, obviously, there’s some sort of integrated way of doing things that being at the hospital is learning. And the doctors try and teach you that, but it doesn’t always seem to hit. So it’s very hard to be at the hospital and be present and try and get involved in things and try to learn what being an intern is like, but then also have this big expectation that, like, you should be reading all the time, you should be studying all the time. Like, you just felt too spread, you know.” (P6 – interview) (ER+)*

Meanwhile the attributes that are most needed in internship are not generally rewarded in the undergraduate space, such as efficiency, teamwork and maintaining a work life balance (weaker social relations: SR–):

*“I think it’s an overlooked thing… I think there’s certain people in my group who are struggling with that and don’t realise why they’re struggling with that, because now it just requires a whole new set of behaviours that they were never really rewarded or encouraged to display as med students.” (P5 – interview) (SR–)*

The graduates best prepared for internship managed to find a balance between performing well enough academically to meet the university requirements, while being self-directed in developing the other professional competencies they needed for internship, representing an elite code (ER+, SR+). This shift in culture was described by participant 9:

*The undergraduate culture was very competitive, almost adversarial, with individual performance and marks being valued. Yet the ability to collaborate, work in a team, communicate when you are struggling, ask for help and look out for each other are critical to succeed in internship (P9*, *informal conversation)*.

#### The Specialisation Plane – summary of results

**Figure 1:**
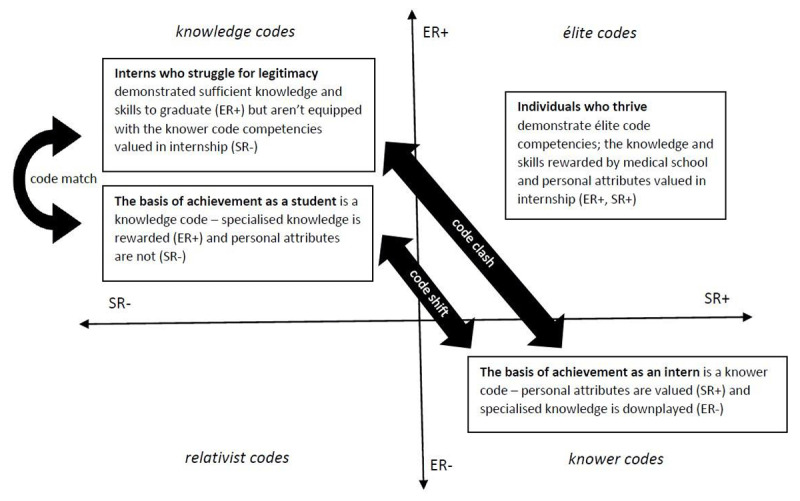
The specialization plane revealing the code shift between medical school and internship. Individuals who struggle for legitimacy in internship have knowledge code dispositions that match with medical school values but clash with the basis of achievement in internship [31].

When these results are plotted on the Specialisation Plane (Figure 1 below), it is clear to see that there is a code shift in the basis of achievement between the knowledge code (ER+, SR–) basis in medical school and the knower code (ER–, SR+) basis in internship. Individuals who are legitimised demonstrate elite code competencies (ER+, SR+) enabling them to successfully navigate the change in the ‘rules of the game’ between medical school and internship. Individuals who find the transition very difficult are those who, while they may have demonstrated enough medical knowledge to qualify, are not equipped with the personal attributes that are valued by the clinical teams in which they are working (ER+, SR–).

## Discussion

In this study we used focused ethnography, grounded in Legitimation Code Theory (LCT), to explore the complex day-to-day reality of the internship role in South Africa and the competencies that form the basis of achievement, building on the existing work readiness literature. Our findings reveal that the role is about managing the demands of executing senior doctors’ plans in a fast-paced environment, where teamwork, organization, efficiency, and adaptability are critical for success. These competencies, legitimized by the clinical community, contrast with the emphasis on specialised medical knowledge in undergraduate training, leaving many interns feeling underprepared for the practical demands of their new role. Our findings add to existing knowledge about why some new interns thrive while others just survive by making explicit the types of competencies that are needed for an intern to succeed in this context. Using focused ethnography, this study extends current knowledge by connecting participants’ narratives with what they actually do, revealing links between how interns describe their experience of internship and their approach to the job and the set of skills and behaviours they demonstrate.

The role of an intern is an important and challenging one, but not necessarily in the ways graduates expected. In this setting the pressures of the job do not come as much from making high-stakes clinical decisions, but rather from being the ‘engine room’ of the hospital, responsible for carrying out management plans in a busy and demanding working environment. Monrouxe et al. [32] suggest much of the difficulty of being a newly qualified doctor comes from the mentally and physically demanding nature of needing to be in multiple places at once and the long hours of hard work with little opportunity to rest. Kellet et al. [33] highlight time management, prioritizing tasks and the large administrative component of the role as particular challenges facing junior doctors. An added pressure we found in our context was the need to regularly and quickly adapt to new systems and working environments where ‘getting the job done’ is a requirement from day one, unlike other settings where newly qualified doctors may have time to find their feet [34]. In order to meet this challenge, a set of personal and social competencies are essential for legitimate practice, including teamwork, organisation, efficiency and communication skills. These competencies are reflected in what de Villiers at al. [35] found supervisors look for in South African interns — conscientiousness and the ability to cope with the workload. While proficiency regarding knowledge and skills was important for each individual’s clinical confidence, interns were surprised by the lack of emphasis placed on the demonstration of complex medical knowledge when it came to being legitimised as a member of the clinical team. Other members of the workplace team ultimately either affirm or disaffirm an intern’s identity [19], so the ability to demonstrate these valued attributes is essential in developing legitimacy in their new intern role.

Our participants recognised that the competencies most legitimised in internship were not highly valued in medical school. Instead, the emphasis was on the demonstration of specialised medical knowledge in high-stakes assessments. Surmon et al. [36] suggest that competencies such as organisation, teamwork and resilience can be developed through appropriate experiences, however, while students face competing internship readiness and medical school priorities, many will focus on the immediate pressures of passing high stakes assessments at the expense of gaining those valuable experiences [4]. This study reveals a shift in what counts between medical school and internship, a change in the ‘rules of the game’ that graduates have to navigate. Undergraduate assessment practices generally reward individual demonstrations of knowledge, yet the basis of achievement in internship is about *who you are* rather than *what you know*. This abrupt change in what matters between being a student and a junior doctor can have a deleterious impact on graduates’ confidence and ability to succeed [5]. Students who focus only on doing well in medical school – getting good grades – can find themselves not as well prepared for internship as they may have expected [11].

While many participants found the transition difficult, some coped well despite the challenges. Two narratives emerged: interns who thrived and those who merely survived. Thriving interns exhibited a wider range of competencies, confidently applying knowledge and demonstrating valued personal attributes. Padley et al. [12] emphasise that confidence in one’s capacity is critical to the transition to clinical practice but this needs to be developed through meaningful clinical roles as students. These interns were positive about internship and their work-life balance, displaying strong organisation, efficiency, and collaboration skills, all skills Sturman et al. [4] highlight as key to workplace transition. As students, these participants actively sought learning opportunities, such as shadowing interns and engaging in patient care, despite limited undergraduate encouragement. The surviving interns were the ones who described themselves as overwhelmed, exhausted and unable to maintain a work-life balance. The skills they struggled with were abilities such as efficiency and time management. They often left late in the day, rarely rested on call and were constantly tired, negatively impacting their overall wellbeing. If others had to step in and pick up the slack, they could quickly become delegitimised within the clinical team.

Using the LCT Specialisation framework, this study shows that while clinical knowledge and skills remain important, personal attributes such as teamwork and communication skills are currently undervalued in the undergraduate space. These attributes form the basis of achievement in the internship setting and are key determinants of legitimacy within the clinical team. This highlights a pressing need for a more holistic conceptualisation of Competency Based Medical Education (CBME) that legitimises all the attributes needed to thrive in the junior doctor role.

### Recommendations for practice

CBME frameworks need to meaningfully integrate and value the competencies that provide legitimacy in internship. Curricula should reward both specialised knowledge and skills and the personal attributes needed for successful practice through constructively aligned learning opportunities and assessments [36]. The need to develop skills such as prioritisation, time management and self-care must not only be made explicit to students but can be actively coached through workshops and mentorship programmes [37]. Maintaining a work-life balance and having time to spend in the clinical space can be made possible by the students not being under constant pressure from high stakes assessments [38]. A programme of fit-for-purpose, workplace-based assessments that reflect holistic outcomes [23] should instead make it clear that spending time in the clinical setting, familiarising themselves with the healthcare system and learning from patients and members of the interprofessional team is what will equip them to not only succeed in internship, but also in medical school [32]. Students need earlier clinical exposure, and in order for them to better understand the responsibility that awaits them, there needs to be a culture shift in the clinical space making undergraduate students legitimate members of clinical teams with defined roles. [39] They should be involved in authentic, practice-based learning of all aspects of patient care, learning not only from senior clinicians but also by shadowing the internship doctors inhabiting the role they are being prepared for [39]. Until the competencies that form the basis of achievement in internship are explicitly valued in the undergraduate curriculum alongside clinical knowledge and skills, many graduates will not be ready for the realities of work.

### Limitations and recommendations for future studies

It was important to be aware of the potential effect that ‘participant reactivity’ – behaviour adaptation that occurs as a result of being observed [30] may have had on the data collected. In the clinical setting this may be limited by demands of care often being too great for providers to alter behaviour in normative ways [30]. When the work is routine, automatic responses are likely; when it is not, focus is likely to be on the task. To further mitigate participant reactivity, the principal researcher conducted prolonged engagements and sustained observations during which they embedded themselves in the environment and attempted to establish trust and rapport with participants. The researcher cross checked their own understandings with the views of the participants during the informal conversations and interviews.

This study took place in one facility, which may limit generalisability to other contexts. Notably, the interns in this study were very well supervised, which is not always the case in the South African context [11]. One of the strengths of this study is that, while the participants were all at one facility, they represented multiple different medical schools. One of the limitations of many graduate outcome studies has been that the majority report on evaluations of single educational institutions [12]. The data collection was done by only one researcher (SP) which may have influenced the findings based on their own ideas and experiences. As participants were self-selected, we may have missed experiences from individuals less motivated to participate.

While our research sheds light on the frictions between what has been learned at the undergraduate level and the types of competencies legitimised in clinical practice, there is still a need to explore, in greater depth, the personal development journey individuals undergo as they transition from student to doctor [12]. To achieve this, perspectives from longitudinal research designs, of which there are very few, are needed to create a clearer picture of the transition [40]. Our future research will aim to answer this call.

## Conclusion

The reality of the internship doctor’s role is that it’s about managing a demanding workload in a fast-paced environment where teamwork, organization, efficiency, and good communication skills are critical for success. Interns who demonstrate these competencies are legitimised within the community, whereas interns who don’t become overwhelmed, leading to stress, fatigue and an inability to maintain a work-life balance. The basis of achievement in internship contrasts with the emphasis placed on specialized medical knowledge in undergraduate training, resulting in many graduates underprepared to meet the expectations of their new role. We need to ensure that our undergraduate curricula explicitly value the personal and social competencies that are critical for legitimate practice in internship.

## References

[1] Prentice S, Dorstyn D, Benson J, Elliott T. Burnout Levels and Patterns in Postgraduate Medical Trainees: A Systematic Review and Meta-Analysis. Acad Med. 2020;95:1444–54. DOI: 10.1097/ACM.0000000000003379 32271234

[2] Carlsson Y, Bergman S, Nilsdotter A, Liljedahl M. The medical internship as a meaningful transition: A phenomenographic study. Med Educ. 2023;57:1230–8. DOI: 10.1111/medu.15146 37283081

[3] Hariharan TS, Griffin B. A review of the factors related to burnout at the early-career stage of medicine. Med Teach. 2019;41:1380–91. DOI: 10.1080/0142159X.2019.1641189 31345077

[4] Sturman N, Tan Z, Turner J. “a steep learning curve”: Junior doctor perspectives on the transition from medical student to the health-care workplace. BMC Med Educ. 2017;17:1–7. DOI: 10.1186/s12909-017-0931-2 28549459 PMC5446747

[5] Maton K, Tsai-Hung Chen R. Specialization codes: Knowledge, knowers and student success. In: Martin JR, Maton K, Doran YJ, editors. Accessing Academic Discourse. 1st ed., New York: Routledge; 2020. pp. 35–58. DOI: 10.4324/9780429280726-2

[6] Touchie C, ten Cate O. The promise, perils, problems and progress of competency-based medical education. Med Educ. 2016;50:93–100. DOI: 10.1111/medu.12839 26695469

[7] Alharbi NS. Evaluating competency-based medical education: a systematized review of current practices. BMC Med Educ. 2024;24:1–10. DOI: 10.1186/s12909-024-05609-6 38831271 PMC11149276

[8] Hamza DM, Hauer KE, Oswald A, van Melle E, Ladak Z, Zuna I, et al. Making sense of competency-based medical education (CBME) literary conversations: A BEME scoping review: BEME Guide No. 78. Med Teach. 2023;45:802–15. DOI: 10.1080/0142159X.2023.2168525 36668992

[9] Ras T, Stander Jenkins L, Lazarus C, van Rensburg JJ, Cooke R, Senkubuge F, et al. “We just don’t have the resources”: Supervisor perspectives on introducing workplace-based assessments into medical specialist training in South Africa. BMC Med Educ. 2023;23:1–10. DOI: 10.1186/s12909-023-04840-x 37932732 PMC10629100

[10] Mnguni L. The Curriculum Ideologies Underlying the AfriMEDS Curriculum Framework for Undergraduate Medical and Dental Education in South Africa. Int Med Educ. 2024;3:44–61. DOI: 10.3390/ime3010005

[11] Pattinson SR, Savelberg H, Atherley A. Not ready in the ways that count– a qualitative exploration of junior doctor’s perceived preparedness for practice using Legitimation Code Theory. Adv Heal Sci Educ. 2025;30:795–814. DOI: 10.1007/s10459-024-10380-w PMC12119643 39373869

[12] Padley J, Boyd S, Jones A, Walters L. Transitioning from university to postgraduate medical training: A narrative review of work readiness of medical graduates. Heal Sci Reports. 2021;4. DOI: 10.1002/hsr2.270 PMC8025846 33855193

[13] Bleakley A, Brennan N. Does undergraduate curriculum design make a difference to readiness to practice as a junior doctor? Med Teach. 2011;33:459–67. DOI: 10.3109/0142159X.2010.540267 21609175

[14] Goodson L, Vassar M. An overview of ethnography in healthcare and medical education research. J Educ Eval Health Prof. 2011;8:4. DOI: 10.3352/jeehp.2011.8.4 21637319 PMC3100516

[15] Atkinson P, Pugsley L. Making sense of ethnography and medical education. Med Educ. 2005;39:228–34. DOI: 10.1111/j.1365-2929.2004.02070.x 15679691

[16] Monrouxe LV, Grundy L, Mann M, John Z, Panagoulas E, Bullock A, et al. How prepared are UK medical graduates for practice? A rapid review of the literature 2009-2014. BMJ Open. 2017;7. DOI: 10.1136/bmjopen-2016-013656 PMC5253586 28087554

[17] Andreassen P, Christensen MK, Møller JE. Focused ethnography as an approach in medical education research. Med Educ. 2020;54:296–302. DOI: 10.1111/medu.14045 31850537

[18] Reeves S, Peller J, Goldman J, Kitto S. Ethnography in qualitative educational research: AMEE Guide No. 80. Med Teach. 2013;35. DOI: 10.3109/0142159X.2013.804977 23808715

[19] Sheehan D, Wilkinson TJ. Widening how we see the impact of culture on learning, practice and identity development in clinical environments. Med Educ. 2022;56:110–6. DOI: 10.1111/medu.14630 34433232

[20] Varpio L, Paradis E, Uijtdehaage S, Young M. The Distinctions Between Theory, Theoretical Framework, and Conceptual Framework. Acad Med. 2020;95:989–94. DOI: 10.1097/ACM.0000000000003075 31725464

[21] Hodson N. Landscapes of practice in medical education. Med Educ. 2020;54:504–9. DOI: 10.1111/medu.14061 31953968

[22] Cruess RL, Cruess SR, Steinert Y. Medicine as a Community of Practice: Implications for Medical Education. Acad Med. 2018;93:185–91. DOI: 10.1097/ACM.0000000000001826 28746073

[23] Sims D, Zingela Z, Mokhachane M, Botha G, Mawela D, Singaram V, et al. Medical education, reflections and perspectives from South Africa: a review. BMC Med Educ; 2025. DOI: 10.1186/s12909-025-06910-8 PMC11900656 40075353

[24] Dubale BW, Friedman LE, Chemali Z, Denninger JW, Mehta DH, Alem A, et al. Systematic review of burnout among healthcare providers in sub-Saharan Africa. BMC Public Health. 2019;19. DOI: 10.1186/s12889-019-7566-7 31510975 PMC6737653

[25] Naidu K, Torline JR, Henry M, Thornton HB. Depressive symptoms and associated factors in medical interns at a tertiary hospital. South African J Psychiatry. 2019;25:1–9. DOI: 10.4102/sajpsychiatry.v25i0.1322 PMC6620542 31308973

[26] Braun V, Clarke V. To saturate or not to saturate? Questioning data saturation as a useful concept for thematic analysis and sample-size rationales. Qual Res Sport Exerc Heal. 2021;13:201–16. DOI: 10.1080/2159676X.2019.1704846

[27] Malterud K, Siersma VD, Guassora AD. Sample Size in Qualitative Interview Studies: Guided by Information Power. Qual Health Res. 2016;26:1753–60. DOI: 10.1177/1049732315617444 26613970

[28] Braun V, Clarke V. One size fits all? What counts as quality practice in (reflexive) thematic analysis? Qual Res Psychol. 2021;18:328–52. DOI: 10.1080/14780887.2020.1769238

[29] Kiger ME, Varpio L. Thematic analysis of qualitative data: AMEE Guide No. 131. Med Teach. 2020;42:846–54. DOI: 10.1080/0142159X.2020.1755030 32356468

[30] Paradis E, Sutkin G. Beyond a good story: From Hawthorne Effect to reactivity in health professions education research. Med Educ. 2017;51:31–9. DOI: 10.1111/medu.13122 27580703

[31] Maton K. Knowledge and Knowers: Towards a realist sociology of education. London: Routledge; 2014. DOI: 10.4324/9780203885734

[32] Monrouxe LV, Bullock A, Gormley G, Kaufhold K, Kelly N, Roberts CE, et al. New graduate doctors’ preparedness for practice: A multistakeholder, multicentre narrative study. BMJ Open. 2018;8. DOI: 10.1136/bmjopen-2018-023146 PMC6119440 30158236

[33] Kellett J, Papageorgiou A, Cavenagh P, Salter C, Miles S, Leinster SJ. The preparedness of newly qualified doctors – Views of Foundation doctors and supervisors. Med Teach. 2015;37:949–54. DOI: 10.3109/0142159X.2014.970619 25308805

[34] Carlsson Y, Nilsdotter A, Bergman S, Liljedahl M. Junior doctors’ experiences of the medical internship: a qualitative study. Int J Med Educ. 2022;13:66–73. DOI: 10.5116/ijme.6229.d795 35321942 PMC9017508

[35] de Villiers M, van Heerden B, van Schalkwyk S. ‘Going the extra mile’: Supervisors’ perspectives on what makes a ‘good’ intern. South African Med J. 2018;108:852–7. DOI: 10.7196/SAMJ.2018.v108i10.13052 30421714

[36] Surmon L, Bialocerkowski A, Hu W. Perceptions of preparedness for the first medical clerkship: A systematic review and synthesis. BMC Med Educ. 2016;16:1–11. DOI: 10.1186/s12909-016-0615-3 26968816 PMC4788861

[37] Picton A. Work-life balance in medical students: self-care in a culture of self-sacrifice. BMC Med Educ. 2021;21:1–12. DOI: 10.1186/s12909-020-02434-5 33407370 PMC7786898

[38] Dornan T, Gillespie H, Armour D, Reid H, Bennett D. Medical students need experience not just competence. BMJ. 2020;371:1–2. DOI: 10.1136/bmj.m4298 33184038

[39] Lulua DR, Moch S. Symbolic access: medical students’ awareness of institutional culture and its influence on learning, a phenomenographic study. BMC Med Educ. 2024;24:1–12. DOI: 10.1186/s12909-023-05001-w 38178107 PMC10768196

[40] Atherley A, Teunissen P, Hegazi I, Hu W, Dolmans D. Longitudinal exploration of students’ identity formation during the transition from pre-clinical to clinical training using research poetry. Med Educ. 2023;57:637–47. DOI: 10.1111/medu.14998 36460437

